# Food Allergy--Lessons from Asia

**DOI:** 10.1097/WOX.0b013e31817b7431

**Published:** 2008-07-15

**Authors:** Bee Wah Lee, Lynette Pei-Chi Shek, Irvin Francis A Gerez, Shu E Soh, Hugo P Van Bever

**Affiliations:** 1 Department of Paediatrics, Yong Loo Lin School of Medicine, National University of Singapore, Singapore; 2 Children's Medical Institute, National University Hospital, Singapore

**Keywords:** food allergy, Asia, shellfish allergy, peanut allergy, buckwheat, bird's nest, chickpeas, royal jelly

## Abstract

**Objective:**

This is a review on published data available on food allergy in East Asia and a discussion on the insights that it offers.

**Methods:**

PubMed searches were made for terms *food allergy *and *anaphylaxis*, in combination with *Asia*.

**Results:**

There is a paucity of population-based prevalence studies on food allergy in Asia. Certain unique food allergens, such as buckwheat, chestnuts, chickpeas, bird's nest, and royal jelly, which are consumed extensively by certain Asian populations have resulted in clinical food allergy of little importance in other populations. Crustacean shellfish is of importance in this region relative to other common food allergens. The high consumption of these foods and possibly coupled with cross-reactive tropomyosins from dominant inhalant dust mite and cockroach allergens in this region may explain this phenomenon. In contrast, the prevalence of peanut allergy is relatively low in this region. The reasons for this difference are not apparent. However, this may be a reflection of the general reduced propensity in this region to allergic diseases as seen with asthma.

**Conclusions:**

Further research on food allergy in Asia is warranted because it offers unique opportunities to further our understanding on the influence of population and environment.

## 

The allergy epidemic has resulted in a global rise in prevalence of allergic diseases such as asthma, allergic rhinitis, and eczema in the recent decades. This increase is highest in affluent communities adopting a westernized lifestyle, with the western populations documenting the highest prevalence [1]. Similar trends have been observed for food allergy, particularly peanut allergy,[2] but these are less well documented globally. The reason, at least in part, may be related to the inherent difficulties ascertaining a population's prevalence of true food allergy. Self-reported food allergy notoriously overestimates the true prevalence, probably because of subjective bias. Nonetheless, such observations between populations are useful starting points for understanding differences in the patterns of food allergy and possibly their underlying mechanisms. The objective of this article is to review the data available on food allergy (immunoglobulin E [IgE] mediated) in East Asia and discuss the insights that it might offer.

## Methods

PubMed searches were made for *food allergy *and *Asia*. In addition, a search was also made for *food allergy *and the respective East Asian countries, China, Japan, Korea, India, Indonesia, Malaysia, Singapore, Philippines, Thailand, and Vietnam. Similarly, these regions were searched in combination with the term *anaphylaxis*.

## Population Studies

There are few population-based studies on food allergy in Asia. The epidemiology of food allergy in Asia has been reviewed recently [3]. Population prevalence studies based on self-reported questionnaires targeted at children younger than 12 years ranged from 4% in Singapore[4] and rural China[5] to as high as 12% in Seoul, Korea,[6] and Japan [7]. This wide range in prevalence mirrors those reported in a recent meta-analysis obtained from surveys on populations elsewhere (3%Y35%),[8] and it was concluded that these differences were related to survey methodologies rather than a true difference. The authors recommended that standardized methods, including specific IgE measurements with standardized allergen extracts, and if possible challenge testing, are necessary to obtain more accurate estimates of prevalence. It therefore seems that comparing the true prevalence of food allergies across populations will still require considerable research effort.

## Food Allergens Unique to Asia

It has been observed that certain specific foods consumed mainly in the Asian region have resulted in allergies that are unique to their respective populations. Allergy to edible bird's nest from swiftlets has been described in the Chinese population in Singapore,[9,10] Malaysia,[11] and Hong Kong (G. W. K. Wong, MD, oral communication, 2006). It is the most common cause of anaphylaxis in Singapore children [12]. This food is a popular Chinese delicacy believed to have health benefits. A salivary protein with homology to the ovomucoid inhibitor has been identified as the major allergen [13,14]. Similarly, royal jelly, another food supplement very popular among the Chinese, has also been reported to trigger asthma and anaphylaxis in Hong Kong[15] and ethnic Chinese in Australia [16].

Buckwheat causing anaphylaxis has been observed in Japan, Korea, and China [17-19]. In a national survey in Japan, it has been ranked fourth as a cause of immediate hypersensitivity [20]. Buckwheat is consumed in large quantities by these populations early large exposure rather than conventional strict avoidance in the form of noodles or soba. Similarly, chickpeas, a staple food in children living in India,[21,22] and chestnuts in Korea[23] have been described as common triggers of immediate hypersensitivity in these populations. These patterns of food allergies in populations of East Asia are not commonly recognized elsewhere. With increasing globalization, it would be necessary for practicing allergists to be familiar with these potential food allergens. It is more likely that exposure rather than genetic factors are responsible for these observations. These studies suggest that unless there is widespread exposure to potential allergenic foods in a population, clinical allergy to such foods may not be apparent.

## Fish Allergens

The fish from tropical waters consumed in Asia are quite different from temperate fish. Consumption practices are also quite different. Fish is a weaning food among many populations in Asia. This contrasts with the western diet, where fish is regarded as a highly allergenic food. Fish allergy affects up to 3% of children in Scandinavian populations,[24] and until very recently, the American Academy of Pediatrics had recommended that fish be avoided until the age of 3 years [25]. There is an impression that fish allergy in this part of the world is less common than the western world, but this has not been substantiated by a systematic study. Of the temperate fish, the major allergen of cod, Gad c 1, belonging to the protein family of parvalbumins, has been the most extensively studied [26]. Evaluation of other temperate fish has shown that parvalbumin constitutes the major cross-reactive fish allergen of these fish [27,28]. The evaluation of 4 species of tropical fish (threadfin, pomfret, Indian anchovy, and tengiri) commonly consumed in Singapore and often used as weaning foods in infants has also shown that parvalbumin is the major allergen of these tropical fish. These parvalbumins are cross-reactive with Gad c 1,[29] which was also clinically evident because most fish-allergic children in this study had clinical reactions to more than 1 fish. Only one of the 10 children evaluated was monosensitized and could tolerate consuming other fish without a clinical reaction. Hence, the allergenicity of tropical fish is comparable with cod. The reason(s) that fish allergy is not highly prevalent in tropical Asia despite high consumption and exposure in early life is not obvious, although it is tempting to postulate that paradoxically, has induced immune tolerance.

## Peanut Allergy

Peanut allergy is recognized as an important food allergy because it is known to cause severe life-threatening reactions, is long-lasting, and is increasingly common in some populations. To date, there have been no published reports on population-based prevalence studies on peanut allergy in Asia, but the impression among clinicians practicing in Asia is that it is uncommon. In Singapore, a peanut allergy survey was conducted on 14-to 15-year-old schoolchildren in 2005, and results were presented at the World Allergy Congress 2007 [30]. These data and those from published studies in the western populations are shown in Table 1. The prevalence of peanut allergy in children in several western populations, such as the United States,[31] United Kingdom,[32-34] Canada,[35] and France[36] are near or slightly higher than 1%. In fact, increasing trends in the US and United Kingdom populations have been documented, and it has been suggested that these populations are experiencing a peanut allergy epidemic [2]. In contrast, the Singapore study that was based on the same questionnaire developed by Sicherer et al[31] for convincing food allergies (with immediate symptoms within 2 hours and typical acute allergic symptoms), the prevalence of peanut allergy was 0.3%, or about a third of those reported in these western populations [30]. The prevalence would be even lower if skin prick tests (SPTs) of a subcohort in the Singapore study are taken into account because only one of the 22 subjects (about 4% with convincing allergies) had a positive SPT. The true prevalence of peanut allergy in Singapore children is therefore likely to be much lower than the 0.3% estimate from the questionnaire study alone.

**Table 1:** Prevalence of Peanut Allergy in Various Population-Based Surveys

Country	Prevalence%, 95% Confidence Interval	No. Surveyed, Response Rate	Age of Population, yrs	Study Year	Reference
United States	0.4*	2998	< 18	1997	Sicherer et al[59]
	0.8*	2948	< 18	2002	Sicherer et al[31]
United Kingdom	1.2**	1218/1456	4	1993-1994	Tariq et al[32]
	1.5**	1273/2878	3-4	1997-1999	Grundy et al[33]
	1.8** (1.1-2.7)	957	4-5	2003-2005	Hourihane et al[34]
Canada	1.5** (1.2-1.9)	4339/7768	7	2000-2005	Kagan et al[35]
France	1	33,110/44,000	< 0-60	1997	Kanny 2001[36]
Singapore	0.3, 0.014**	6765/8072	14-15	2005-2006	Gerez et al[30]

*Based on nationwide telephone survey with standardized questionnaire.

**Taking into account SPTs, for Singapore study only done in a subcohort.

The notion that peanut allergy is not as prevalent in Asia is also substantiated by hospital-based studies on anaphylaxis. Studies from Singapore in children[12] and adults[10] have shown that severe peanut allergy resulting in anaphylaxis was very uncommon, with no documented cases in children and only 2 adult patients (2.7%). These data are corroborated by a study on anaphylaxis from Thailand (adults and children), where no cases of peanut allergy were recorded [37]. Similarly, a study from Hong Kong reported crustacean shellfish as the most common food trigger in 89% of cases, but there was no mention of peanut allergy [38]. The data from Asia contrast markedly with those from the United States,[39] United Kingdom,[40] and Australia,[41] where hospitalization and fatalities caused by peanut allergy are well documented.

This apparent low prevalence rate of peanut allergy in Asian populations is not likely caused by lack of exposure. Sensitization to peanuts is not uncommon among the atopic population here. In a hospital-based study of children older than 3 years attending a hospital-based allergy clinic in Singapore, sensitization to peanuts rates 27.3% and is ranked third most common after egg and shellfish,[42] and in a separate study, the rate was 12% in those younger than 3 years [43]. Relatively high rates of sensitization have also been recorded among allergic Hong Kong (31% in atopic eczema)[44] and Taiwanese children (36% in atopic eczema) [45].

The data presented here strongly support the notion that the low prevalence of peanut allergy in Asian populations compared with that in North America and the United Kingdom is true and not just conjecture. In view of the relatively high peanut sensitization among the atopic subjects in these populations, this low prevalence of allergy in Asia is more likely caused by immune tolerance rather than the lack of exposure. Resolving the reasons for this difference between populations would provide important insights into possible public health measures that could prevent further escalation of the peanut allergy epidemic of the West. Speculation on environmental and genetic differences has been discussed in detail in a recent review by Sicherer and Sampson [2]. The reason regarding the method of cooking and the allergenicity of roasted peanuts compared with boiled peanuts[46] is an often mentioned reason, although roasted peanut such as peanut butter is also widely available in Asia. Environmental rather than genetic factors are likely to play an important role in these differences in the prevalence of peanut allergy between geographic regions because the data from the United States suggest that peanut allergy prevalences of all ethnicities are similar [31].

## Crustacean Shellfish Allergy

In contrast to the low prevalence of peanut allergy in Asia, crustacean shellfish seems to be an important cause of food allergy. In terms of severity, hospital-based studies on anaphylaxis show that crustacean shellfish are one of the most important food triggers in adults and children in Singapore[10,12] and Thailand,[37] and adults in Hong Kong [38]. Interestingly, this phenomenon seems to be reversed in western populations, with less severe crustacean shellfish allergy in comparison with peanut allergy. Only a few or no cases of crustacean shellfish-induced anaphylaxis were reported in hospital-based surveys in children in the United Kingdom,[47] Italy,[48] and children and adults in Australia [49]. Instead, peanut-triggered anaphylaxis predominates in these populations. In addition, registries recording fatalities caused by anaphylaxis in the United Kingdom[40] and United States[39] also show that although peanuts and tree nut are important triggers, there was only 1 case triggered by crustacean seafood in the United Kingdom registry. It therefore seems that there is a reversal of importance between these food allergens-- the importance of peanut in the West and crustacean shellfish in Asia.

Based on population surveys, the prevalence of crustacean shellfish allergy in the United States for all ages is 2%, and 0.1% for the 0-to 5-year and 0.8% for the 6-to 17-year age group [50]. Slightly lower prevalence rates were found in Denmark. This study involved a birth cohort and their family members, which showed the absence of shellfish allergy in children (up to 21 years) and a prevalence of 0.3% in adults [51]. Like peanut allergy, there have been no published studies on population surveys in any Asian population. However, a survey conducted in Singapore schoolchildren aged 14 to 15 years presented as an abstract showed that the positive responses for convincing shellfish allergy are comparatively high (3.95% or about 5 times that of US children),[52] although most had mild symptoms (L.P-C.S., unpublished data, 2007).

Like fish, crustacean shellfish is a major component of the East Asian diet. However, unlike fish allergy, this increased exposure may explain the high prevalence of shellfish allergy in this region. Sensitization to shrimp has been observed in our atopic population from an early age. The sensitization rate was reported to be 3.6% in atopic children younger than 1 year, and 10.6% in those between 1 and 3 years [43]. Because exposure to fish and peanuts has not resulted in a high prevalence of allergy to these food allergens in Asia, it is tempting to speculate an alternative hypothesis for the high prevalence of shellfish allergy. The high prevalence of inhalant dust mite and cockroach allergies in tropical and subtropical Asia may contribute to cross-reacting allergens through the panallergen tropomyosin [53]. This protein is a major allergen in shrimp and shellfish allergy. If confirmed, this would be akin to the pollen-food syndrome observed in the temperate climates,[54] and the cross-reacting tropomyosin allergens may be responsible for the mild form of shellfish allergy, very much like the described oral allergy syndrome. This hypothesis is supported by the corelation of sensitization to shrimp and cockroach allergens in Singapore children,[42] as well as population studies on unexposed Jews who observed Kosher dietary rules, which showed that sensitization to shrimps was related to cross reacting tropomyosin allergens in house-dust mites [55]. Furthermore, in a study of 17 house dust mite allergic (HDM) patients receiving immunotherapy, 3 developed IgE against shrimp, and 2 of these having IgE against tropomyosin had oral allergy symptoms after ingesting shrimp [56].

## Concluding Remarks

The data on food allergy in Asia have provided insight into the relative importance of certain food allergens in this region. Foods such as buckwheat, chestnut, chickpea, bird's nest, and royal jelly, which are consumed extensively within certain Asian populations, have resulted in clinical food allergy that is of little or no importance in other populations. Crustacean shellfish also seems to be of importance relative to other common food allergens. Whether high consumption of shellfish coupled with cross-reactive tropomyosins from inhalant dust mite and cockroach allergens (inhalant food/oral allergy syndrome) is responsible for these observations deserves further evaluation. In contrast, peanut allergy and anaphylaxis triggered by peanuts is relatively low in this region. This may reflect the relatively generally reduced propensity to allergic diseases in these populations, as is seen with asthma [57]. However, unlike the rising trends in asthma in the urbanized Asian communities, similar increases in peanut allergy are not yet apparent. Although the reasons for this are not obvious, the possibility that the threshold of developing inhalant allergies is lower than that for food allergies may be a consideration, as there is efficient induction of immune tolerance via the gastrointestinal tract [58].

## End note

Supported by the National Medical Research Council Singapore (grant R-178000-131-112).
